# Transcription Errors of Blood Glucose Values and Insulin Errors in an Intensive Care Unit: Secondary Data Analysis Toward Electronic Medical Record-Glucometer Interoperability

**DOI:** 10.2196/11873

**Published:** 2019-03-25

**Authors:** Azizeh Khaled Sowan, Ana Vera, Ashwin Malshe, Charles Reed

**Affiliations:** 1 School of Nursing University of Texas Health at San Antonio San Antonio, TX United States; 2 Center for Clinical Excellence University Health System San Antonio, TX United States; 3 College of Business University of Texas at San Antonio San Antonio, TX United States

**Keywords:** transcription errors, blood glucose, insulin errors, interoperability, glucometer, electronic medical records, secondary data analysis, intensive care units, medication errors

## Abstract

**Background:**

Critically ill patients require constant point-of-care blood glucose testing to guide insulin-related decisions. Transcribing these values from glucometers into a paper log and the electronic medical record is very common yet error-prone in intensive care units, given the lack of connectivity between glucometers and the electronic medical record in many US hospitals.

**Objective:**

We examined (1) transcription errors of glucometer blood glucose values documented in the paper log and in the electronic medical record vital signs flow sheet in a surgical trauma intensive care unit, (2) insulin errors resulting from transcription errors, (3) lack of documenting these values in the paper log and the electronic medical record vital signs flow sheet, and (4) average time for docking the glucometer.

**Methods:**

This secondary data analysis examined 5049 point-of-care blood glucose tests. We obtained values of blood glucose tests from bidirectional interface software that transfers the meters’ data to the electronic medical record, the paper log, and the vital signs flow sheet. We obtained patient demographic and clinical-related information from the electronic medical record.

**Results:**

Of the 5049 blood glucose tests, which were pertinent to 234 patients, the total numbers of undocumented or untranscribed tests were 608 (12.04%) in the paper log, 2064 (40.88%) in the flow sheet, and 239 (4.73%) in both. The numbers of transcription errors for the documented tests were 98 (2.21% of 4441 documented tests) in the paper log, 242 (8.11% of 2985 tests) in the flow sheet, and 43 (1.64% of 2616 tests) in both. The numbers of transcription errors per patient were 0.4 (98 errors/234 patients) in the paper log, 1 (242 errors/234 patients) in the flow sheet, and 0.2 in both (43 errors/234 patients). Transcription errors in the paper log, the flow sheet, and in both resulted in 8, 24, and 2 insulin errors, respectively. As a consequence, patients were given a lower or higher insulin dose than the dose they should have received had there been no errors. Discrepancies in insulin doses were 2 to 8 U lower doses in paper log transcription errors, 10 U lower to 3 U higher doses in flow sheet transcription errors, and 2 U lower in transcription errors in both. Overall, 30 unique insulin errors affected 25 of 234 patients (10.7%). The average time from point-of-care testing to meter docking was 8 hours (median 5.5 hours), with some taking 56 hours (2.3 days) to be uploaded.

**Conclusions:**

Given the high dependence on glucometers for point-of-care blood glucose testing in intensive care units, full electronic medical record-glucometer interoperability is required for complete, accurate, and timely documentation of blood glucose values and elimination of transcription errors and the subsequent insulin-related errors in intensive care units.

## Introduction

### Background

Glycemic control in critically ill patients is essential to improve clinical outcomes and decrease morbidity and mortality [1-8], specifically for patients admitted to intensive care units (ICUs) for more than 3 days [2] and for patients admitted to surgical trauma ICUs (STICUs) compared with medical ICUs [7]. Critically ill patients require constant point-of-care tests (POCTs) for blood glucose to guide initiation and titration decisions regarding continuous insulin infusion following insulin management protocols. Handheld blood glucose monitoring devices or glucometers are widely used in ICUs for this purpose for convenience and portability [9,10].

Transcribing blood glucose readings from glucometers into a paper log and different flow sheets in the electronic medical record (EMR) by health care professionals is a very common yet error-prone practice in ICUs, given the lack of interoperability or connectivity between glucometers and the EMR in many US hospitals [11]. Interoperability allows for wireless transfer of blood glucose values from glucometers to the EMR without the need for manual data entry. Despite the call for system interoperability and emerging research describing frameworks and prototypes for seamless integration of medical device data into the EMR using different connectivity standards [12-15], medical device-EMR connectivity is limited in the United States. In a national survey of 825 US hospitals, the Health Information and Management Systems Society Analytics team reported a lack of any interface between EMRs and medical devices in 70% of the hospitals. The remaining 30% of the hospitals reported an interface of an average of 2.6 device types (out of 11 devices) with their EMRs. Interestingly, none of the hospitals provided an interface between glucometers and the EMR [11].

Extensive literature exists on the use of glucometers in ICUs. However, most studies focused on the glucometers’ accuracy in comparison with other blood glucose analytical measures [16-24]. Research on transcription errors is also available [25-27]; however, there is a paucity of research on transcription errors of blood glucose values obtained by glucometers into the EMRs and the subsequent insulin errors [28]. Although the use of glucometers with high specificity and sensitivity is essential in critical care settings to prevent harmful effects of erroneous blood glucose readings and the subsequent underdose or overdose of insulin therapy, accurate and instant documentation of blood glucose values obtained by glucometers into the EMR is equally important to inform glycemic control and insulin management decisions.

### Objective

This study examined (1) transcription errors of blood glucose values obtained by a glucometer that were documented in the paper log by technicians and in the EMR vital signs flow sheet by nurses in the ICU, (2) insulin errors resulting from transcription errors of blood glucose values, (3) lack of documenting blood glucose values in the paper log and the EMR vital signs flow sheet, and (4) meter docking time.

## Methods

### Design, Sample, and Setting

This secondary data analysis study examined 5049 blood glucose tests for transcription errors, insulin errors, lack of documenting blood glucose values in the paper log and the EMR, and meter docking time. The study took place in a 30-bed STICU located in a 705-bed university teaching hospital with a large referral base in the southwestern United States. The STICU has an annual admission rate of 1600 patients and an approximate monthly admission rate of 133 patients. At the time of the study, there were 46 full-time and 11 part-time nurses and 13 technicians working in the unit. The average range of blood glucose POCTs performed on patients in the unit is 4200 to 4300 tests per month.

After obtaining institutional review board approval from the University of Texas and the University Health System (number 20140330H), we performed the audit of blood glucose tests and insulin data in a 20% stratified sample of all blood glucose tests available in the meters for patients admitted during 4 months (July to October 2016). Stratification was based on the working shift (day or night) as the only possible factor that may introduce transcription errors of blood glucose readings as a result of fatigue expected at the end of each working shift and on the night shift. Additionally, when we selected a blood glucose test, we also included all blood glucose tests pertinent to the same patient within the same episode of admission to evaluate errors per patient. This resulted in a total of 5049 blood glucose tests.

### Description of the Point-of-Care Testing of Blood Glucose

**Figure 1:**
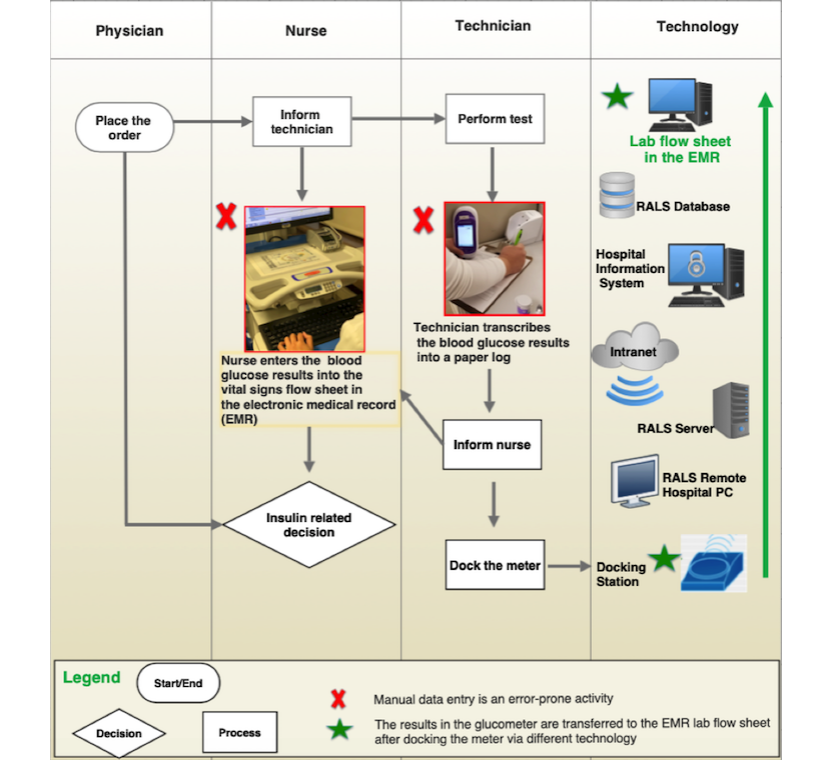
Workflow model of the point-of-care-testing of blood glucose. PC: personal computer.

The point-of-care glucose testing device is Accu-Chek Inform II (Roche Diagnostics Corporation, Indianapolis, IN, USA). Figure 1 depicts a functional workflow model for this process of testing. The process starts by the physician ordering a POCT. The nurse informs the technician about the order, who in turn performs the test using the glucometer and transcribes the result into a paper log—a grid that includes the patient’s name, visit identification number (VIN), room number, time and date of the test, and the result. The VIN is a unique number for each patient episode of admission that is obtained by scanning the patient’s wristband at the time of performing the test.

Nurses then manually enter the readings for each patient into the EMR vital signs flow sheet and use this information to inform their insulin management decisions following physician orders and insulin management protocols. Clinical decisions include whether to continue to monitor, repeat the test to verify critical blood glucose values, inform the physician, give insulin, and titrate the insulin drip based on the insulin management protocol. The blood glucose values entered by nurses into the EMR vital signs flow sheet can be obtained (1) from the glucometer itself by manually searching the readings using the time of the test and the patient VIN to locate the test value, (2) from the technician, who verbally endorses the value to the nurse if he or she is available in the unit, or (3) by checking the value transcribed by the technician into the paper log.

The technician docks the meter by placing it into the meter base unit within 24 hours after the time of the first test for a given day. Meters maintain log data for up to 2000 readings. Since the meter can be docked after 24 hours of use, nurses usually base their insulin management decisions on the readings transcribed by the technicians into the paper log or the readings entered by the nurses into the vital signs flow sheet. By docking the meter, readings are automatically uploaded into the RALS-Plus database, which interfaces with the EMR laboratory flow sheet. These data include the examiner’s employee identification number, patient identification (name, VIN), date and time of the test, time the meter was docked, and blood glucose values. It is worth noting that there is no direct link or seamless transfer of data in the EMR between the vital signs flow sheet and the EMR laboratory flow sheet.

The RALS-Plus v1.5.1 (Alere North America, LLC, Orlando, FL, USA) is a bidirectional interface software for in-hospital glucometers that uploads meter data into the EMR laboratory flow sheet only. The software also generates different types of reports for quality improvement. Data can be generated based on criteria such as the start and end date of the test, blood glucose values, patient VIN, sample type, and test location. Reports can be emailed, printed, saved, or exported into an Excel, rich text (rtf), or pdf file format.

### Main Outcome Variables

#### Transcription Errors

Since the focus of this study was transcription errors, we assumed that technicians follow best practices in obtaining blood samples and in meter use according to the unit policies and procedures and the glucometer’s user manual. Blood glucose values uploaded into RALS-Plus are those in the meters and they are transferred to the EMR laboratory flow sheet. These values are accurate. Transcribing blood glucose values from the meters to the paper log and the EMR vital signs flow sheet may result in 3 potential types of errors (Table 1). The “corresponding values” (Table 1) in the paper log and the EMR vital signs flow sheet are based on the same patient VIN, same date of the test, and within a 1-hour time frame from the POCT (time in RALS) to the time the test was transcribed into the paper log or the EMR vital signs flow sheet.

**Table 1:** Types of errors in transcribing blood glucose values from meters to the paper log and the electronic medical record (EMR) vital signs flow sheet.

Paper log	EMR vital signs flow sheet	
	Flow sheet correct	Flow sheet wrong
Paper correct	*No error:* The blood glucose value in the RALS database matches the corresponding value transcribed by technicians and nurses into the paper log and the EMR vital signs flow sheet for a given test in a given date and time.	*Vital signs flow sheet error:* Any discrepancy regardless of the magnitude between blood glucose value in the RALS database and the corresponding value transcribed by nurses into the EMR vital signs flow sheet.
Paper wrong	*Paper log error:* Any discrepancy regardless of the magnitude between blood glucose value in the RALS database and the corresponding value transcribed by the technician into the paper log.	*Paper log and vital signs flow sheet error:* The 2 blood glucose values transcribed by technicians and nurses into the paper log and the EMR vital signs flow sheet for a given test in a given date and time do not match the value in the RALS database.

#### Undocumented Values of Blood Glucose Tests

Untranscribed or undocumented blood glucose values are those available in RALS database but were not transcribed into the paper log or entered into the EMR vital signs flow sheet.

#### Insulin Errors Related to Erroneously Transcribed Blood Glucose Values

For each transcription error, we also examined whether that error resulted in giving the wrong dose of insulin. We evaluated the wrong insulin dose based on administering a higher or lower insulin dose, regardless of the magnitude of the difference, than the one recommended by the protocol for the correct blood glucose value (the value in the RALS system) or not giving insulin when it should be administered to the patient according to the insulin management protocol based on the correct blood glucose value.

#### Meter Docking Time

As mentioned above, we considered a 1-hour time frame from the POCT (time in RALS or glucometer) to the time the test result was transcribed into the paper log or the EMR vital signs flow sheet when we retrieved the time for transcribing blood glucose values. Meter docking time was retrieved from the RALS database and is the time from the POCT to the time meters were docked (readings were uploaded into the EMR laboratory flow sheet).

In addition to these outcomes, we also collected patient demographics and clinical-related information such as age, sex, diagnosis, diabetes status, admission and discharge dates, and total number of POCTs the patient underwent during the ICU stay.

### Data Collection Procedure

We took the following steps in the sequence identified to collect the data. Three nurse educators collected the data from the paper log and the EMR vital signs flow sheet to enhance objectivity.

First, we accessed the RALS database for the selected study months and downloaded the Excel file (Microsoft Corporation). The file included the patient’s name, VIN, EMR number, test date and time, blood glucose value (meter value), and time of docking the meter.

Second, we selected a stratified sample of 20% of the blood glucose readings and the related information from RALS from the Excel file. In addition to the 20% sample of readings, we went back and selected all pertinent blood glucose tests within the episode of admission for all VINs included in the stratified sample.

Third, for each test selected from RALS, we accessed the EMR and obtained patient demographics and clinical-related information based on the VIN, as well as the corresponding values of blood glucose transcribed into the vital signs flow sheet and time of documentation. We also accessed the laboratory flow sheet to make sure that the tests in RALS were pertinent to that patient.

Fourth, for each test selected from RALS (step 2) for each patient and based on the VIN, we accessed the paper log using the patient’s name and VIN as the identifiers. We obtained the corresponding blood glucose value for each test using the date and a 1-hour time frame from the POCT (time in RALS) as the matching codes. We also obtained the actual time of the test documented in the paper log.

### Data Analysis

We used R statistical computing software v3.5.1 (R Foundation) to analyze the data. Patients’ characteristics and all types of errors were presented using descriptive statistics. We examined the difference in number of POCTs between diabetic and nondiabetic patients using Student *t* test with significance set at *P*<.05.

We limited the analysis of transcription errors to cases where the results of the blood glucose tests were transcribed by clinicians and nurses. For example, the denominator for the paper log transcription errors was the number of blood glucose readings transcribed into the paper log, excluding missing values (ie, when the readings were not transcribed).

## Results

### Patient Characteristics and Number of Point-of-Care Tests

The 5049 blood glucose tests analyzed for transcription errors, undocumented blood glucose readings, and meter docking time were pertinent to 234 unique patients, each with a unique VIN. Table 2 presents the patients’ characteristics. Most of the patients with documented diabetes status in the dataset did not have diabetes (93/234). Of the 234 patients, 97 were with unknown diabetes status. The average number of POCTs performed on diabetic patients (Table 3) was significantly higher than on nondiabetic patients (*t*_47_=–2.17, *P*=.03). One of the patients had 792 POCTs during his stay (Table 3). The median number of POCTs for diabetic patients was 12 tests.

**Table 2:** Patient characteristics (N=234).

Patient characteristic		Statistics
Age in years, mean (SD)		57.5 (17.4)
Length of stay in days, mean (SD)		24.8 (48.3)
Number of point-of-care tests per patient, mean (SD)		25.5 (67.9)
Male sex, n (%)		131 (56.0)
**Diabetes status, n (%)**		
	Yes	44 (32.1)
	No	93 (67.9)
	Missing	97 (41.5)

**Table 3:** Comparison of the number of point-of-care tests between diabetic and nondiabetic patients.

Diabetes status	Minimum	Median	Mean (SD)	Maximum
Diabetes (n=44)	1	12	60 (126)	792
No diabetes (n=93)	1	6	19 (40)	344

### Missing Documentation and Transcription Errors

Table 4 describes the number of tests that were not transcribed into the paper log or the EMR vital signs flow sheet, or both, as well as the number of transcription errors. In the vital signs flow sheet, 40.88% of the tests (2064/5049 tests) were not transcribed. Of the blood glucose tests, 4.73% (239/5049 tests) were not transcribed in the paper log and in the EMR vital signs flow sheet at the same time.

**Table 4:** Number of undocumented blood glucose tests and number of transcription errors among the 5049 tests analyzed.

Source	Undocumented tests, n (%)	Tests analyzed for errors, n	Errors among tests analyzed, n (%)	Range of error, mg/dL (mmol/L)^a^	Error rate per patient (N=234)
Paper log	608 (12.04)	4441	98 (2.21)	–92 to 92 (–5.1 to 5.1)	0.4 (98/234)
Vital signs flow sheet	2064 (40.88)	2985	242 (8.11)	–110 to 80 (–6.1 to 4.4)	1.0 (242/234)
Both	239 (4.73)	2616	43 (1.64)	N/A^b^	0.2 (43/234)

^a^Range of difference between the correct blood glucose value and the erroneously transcribed value.

^b^N/A: not applicable.

We analyzed all types of transcription errors when the blood glucose value was transcribed (Table 4). Of the transcription errors among the 4441 transcribed tests in the paper logs, there were 98 (2.21%) errors. These errors were related to 30 of the 234 patients (12.8%). Of the 2985 transcribed values in the vital signs flow sheet, there were 242 (8.11%) errors related to 63 of the 234 patients (26.9%). The total number of paper log and vital signs flow sheet transcription errors among the 2616 tests analyzed was 43 (1.64%), related to 24 of the 234 patients (10.3%). Overall, among the 234 patients, there were 68 (29.1%) unique patients involved in all types of errors.

Errors in the paper log resulted in transcribing a blood glucose value that was up to 92 mg/dL (5.1 mmol/L) lower or 92 mg/dL (5.1 mmol/L) higher than the correct value (the one in the EMR laboratory flow sheet or RALS). However, most errors, those between the 25th and 75th percentiles, were 12 mg/dL (0.7 mmol/L) lower to 7 mg/dL (0.4 mmol/L) higher than the accurate value. In the EMR vital signs flow sheet, the difference between the correct blood glucose value and the erroneously transcribed value was 110 mg/dL (6.1 mmol/L) lower to 80 mg/dL (4.4 mmol/L) higher. Most errors, those between the 25th and 75th percentiles, were 3 mg/dL (0.16 mmol/L) lower to 4 mg/dL (0.2 mmol/L) higher than the accurate values.

There were no significant differences in the number of transcription errors between the day shift and night shift (Table 5).

**Table 5:** Difference in transcription errors between the day shift and night shift.

Source	Total errors, n (%)	Day shift, n (%)	Night shift, n (%)	Chi-square	*df*	*P* value
Paper log	98/4441 (2.21)	53/2790 (1.90)	45/1651 (2.73)	2.9	1	.09
Vital signs flow sheet	242/2985 (8.11)	163/1847 (8.83)	79/1138 (6.94)	3.1	1	.08
Both	43/2616 (1.64)	24/1639 (1.46)	19/977 (1.94)	0.6	1	.44

### Insulin Errors

The 242 transcription errors in the EMR vital signs flow sheet resulted in 24 insulin errors. These errors resulted in giving 10 U lower to 3 U higher insulin dose than the dose that should have been given had there been no transcription errors. The 98 transcription errors in the paper log resulted in 8 insulin errors and giving 2 to 8 U lower insulin dose than the dose that should have been given had there been no transcription errors. The 43 errors in the EMR vital signs flow sheet and paper logs resulted in 2 insulin errors, both with 2 U lower than the correct insulin dose. Overall, there were 30 unique insulin errors that affected 25 of the 234 patients (10.7%).

### Documentation Time

The average time from the POCT to the time meters were docked (readings were uploaded into the EMR laboratory flow sheet) was 8 hours with a median of 5.5 hours. Most readings, between the first and the third quartiles, took 1.3 to 12 hours to be uploaded into the EMR laboratory flow sheet. Some of the readings took 56 hours (2.3 days) to be uploaded into the EMR laboratory flow sheet.

In addition to these outcomes, we found 40 readings that were documented to some patients’ EMRs and the paper log after the date of discharge.

## Discussion

### Principal Findings

This study examined transcription errors of blood glucose tests obtained by a glucometer and documented in the paper log by ICU technicians and in the EMR vital signs flow sheet by ICU nurses. Insulin errors resulted from transcription errors of blood glucose values, the number of undocumented blood glucose tests in the paper log and the EMR vital signs flow sheet, and the average meter docking time. Research on the use of glucometers in ICU and non-ICU settings is extensive. However, most of these studies focused on precision and accuracy of the glucometers, sources of glucometer measurement errors, and the difference in sensitivity and specificity between glucometer devices from different vendors [16-29]. Nevertheless, glucometers are commonly used handheld devices to measure blood glucose at the point of care, specifically in ICUs to inform timely clinical decisions regarding insulin therapy. To our knowledge, this is the first study to examine transcription errors of blood glucose tests obtained by glucometers and to focus on the urgent need for EMR-glucometer interoperability.

Transcription errors ranged from 2% for paper log errors to 8% for vital signs flow sheet errors. These errors resulted in a total of 30 insulin errors and affected 11% of the patients. The higher percentage of transcription errors in the vital signs flow sheet than in the paper log might be explained by a clinical workflow that has nurses obtain the results of blood glucose tests from 3 different sources, which are the paper log, the technicians, or the glucometers, while the technicians obtain the values only from the glucometers. Transcription errors in the vital signs flow sheet are clinically more significant than transcription errors in the paper log because they inform nurses’ insulin management decisions. These errors affected 63 (27%) patients.

It is important to note that we examined transcription errors and the associated insulin errors only when the blood glucose test results were transcribed by technicians and nurses. The very high percentage of untranscribed values (ie, up to 41% untranscribed into the vital signs flow sheet, n=2064) could mask the actual rate of transcription errors. Possible explanations for not transcribing blood glucose values might be workload issues and the assumption that all readings eventually will be available in the laboratory flow sheet in the EMR after docking the meter. In addition, finding 40 readings documented to some patients’ EMRs and the paper log after the date of discharge is alarming. This means that technicians were not scanning the patient bracelet but probably a sticker that remained on the paper log or the patient monitor or bed. Although eliminating the use of a paper log via a full EMR-glucometer interoperability could decrease this error, adherence to the unit policies and procedures for safe testing is critical for complete elimination of this error.

Although a partial interface exists in our hospital between glucometers and the EMR through the RALS bidirectional interface software, this interface transfers the data only to the EMR central laboratory flow sheet. Additionally, based on the unit policies and procedures, the meters should be docked within 24 hours by technicians. This long time period hinders the availability of the tests’ values at the point of care, making these data unusable for immediate clinical decisions. Furthermore, our results showed that, in reality, docking the meters might take up to more than 2 days. Therefore, there is an urgent need for full glucometer-EMR connectivity to allow for seamless transfer of meter data into other fields of the EMR (ie, the vital signs flow sheet) in order to eliminate data transcription errors and the associated insulin errors.

The few available studies on medical devices-EMR connectivity have focused on vital signs monitors in ICUs and supported improved efficiency and elimination of transcription errors when vital signs monitoring devices are connected to the EMR [14]. The results of our study support the urgent need for a comprehensive and instant connectivity to transfer glucometer data to all fields of the EMR to better inform clinical decisions and eliminate insulin errors associated with transcription errors. On the other hand, from an engineering perspective, interoperability challenges do exist. These may include lack of research describing successes and challenges, the complexity of data elements, and the difference in type of information and formats in which information is stored and displayed. Most important, studies supported the potential for new types of errors in device connectivity, such as transferring the data into the wrong patient’s EMR, in addition to the slow speed of the interface attributed to the slow speed of older medical devices and computers [12]. Therefore, the process of and errors associated with interoperability should be carefully examined.

### Limitations

The results of this study should be interpreted in light of the following limitations. First, since workload, admission rate, and the large number of monthly POCTs are inherent factors that may affect transcription errors, our results can only be generalized to STICUs with a similar workload and rate of POCTs. Second, we limited the errors examined in this study to transcription errors; measurement errors of blood glucose values that may result from inappropriate testing or scanning the wrong patients were beyond the scope of this study. Third, because we collected retrospective data, our risk assessment was limited to identifying the number of insulin errors resulting from transcription errors without identifying the clinical consequences or adverse events of insulin errors. On the other hand, insulin is a high-alert medication and errors in its administration may cause serious hypoglycemia and hyperglycemia, seizures, coma, ketoacidosis, and even death [30].

### Conclusions

Transcription errors of blood glucose values obtained by glucometers do exist and result in insulin errors. Given the high dependence on glucometers for POCTs of blood glucose in ICUs, full EMR-glucometer interoperability is required for complete and accurate documentation of blood glucose values, and elimination of transcription errors and the subsequent insulin-related errors in ICUs.

## Abbreviations

EMR: electronic medical record
ICU: intensive care unit
POCT: point-of-care test
STICU: surgical trauma intensive care unit
VIN: visit identification number
